# Cannabis Users' Recommended Warnings for Packages of Legally Sold Cannabis: An Australia-Centered Study

**DOI:** 10.1089/can.2016.0029

**Published:** 2016-11-01

**Authors:** John M. Malouff, Caitlin E. Johnson, Sally E. Rooke

**Affiliations:** ^1^ Department of Psychology, University of New England, Armidale, Australia.; ^2^ University of Sydney, Sydney, Australia.

**Keywords:** cannabis, health, marijuana, safety, users, warnings

## Abstract

**Introduction:** Although cannabis use creates health risks, governments have recently been legalizing either medical use or leisure use. These governments can mandate health warnings on cannabis packages. Prior research examined recommended warnings of cannabis experts. The aim of this study was to obtain suggested cannabis health and safety warnings from cannabis users.

**Methods:** We used a media release, Facebook postings, and announcements in university classes to seek individuals who had used cannabis at least once according to their own report. Using online data collection software that keeps participants anonymous, we asked the individuals to suggest a warning that governments could mandate on cannabis packages.

**Results:** In total, 288 users suggested warnings. Categorizing the warnings into content categories led to six warning topics: (1) risk of harm to mental health and psychological functioning; (2) risk of operating machinery while under the influence; (3) short-term physical side effects; (4) responsible use; (5) long-term negative physical effects; and (6) dependence, addiction, or abuse. The user-suggested warnings overlapped with six expert-recommended warnings identified in prior survey research and included two content areas that did not feature in expert-recommended warnings: short-term physical side effects and the importance of responsible use.

**Conclusions:** The results are consistent with prior findings that some youths perceive cannabis use as potentially harmful. The current findings provide possible new content for warnings on cannabis packages.

## Introduction

Cannabis legalization is spreading. At present 25 U.S. states and the District of Columbia allow the sale of medical cannabis.^1,2^ Some entire countries permit sale of cannabis for medical purposes; additional states and countries are moving in that direction.^3^ Alaska, Colorado, the District of Columbia, and Washington State now allow the sale of leisure cannabis, and other states are considering following suit.^4^ Uruguay recently became the first nation in modern times to authorize the sale of leisure cannabis.^5^

Cannabis use creates risks to health and safety. The most recent reviews of this evidence^6,7^ indicate that cannabis is associated with (1) *acute effects* that include impaired memory, motor coordination, and judgment; impaired driving; paranoia and psychosis; and (2) *long-term-use effects* of structural changes in brain structures, addiction, withdrawal, including dysphoria, craving, and insomnia, on ceasing use, problems in work, school, and relationships, mental health problems, including anxiety, depression, and psychosis, unemployment, decreased income, criminal behavior, lung disease, heart disease, stroke, and fetal harm. Special risks may arise with use of high-potency (high tetrahydrocannabinol) types of cannabis^7^ and use of edible cannabis.^8^

Causation is not completely clear for all these risks, as third variables, including genetic and other risk factors and the use of other drugs, such as tobacco, might contribute to both cannabis use and some health problems.^7^ These individual differences could explain sudden deaths that seem due to cannabis use.^9^ In addition, the risks of use may vary with specific strains and potencies of cannabis used and with level of use.

Research findings on alcohol warnings suggest that they increase awareness of drinking-related risks.^10^ Studies have shown that smokers consider tobacco warnings effective, that the warnings increase awareness of risks, and that smoking decreases when prominent warnings are introduced.^11^

At present, no nation mandates warnings on legally sold cannabis packages, even though mandated warnings are common on tobacco and alcohol containers.^11,12^ Some U.S. states do mandate specific health and safety warnings on legally sold cannabis. For example, Colorado mandates the following warnings: “There may be health risks associated with the consumption of this product,” “There may be additional health risks…for women who are pregnant, breastfeeding, or planning on becoming pregnant,” “The intoxicating effects of this product may be delayed by two or more hours (for cannabis consumed orally),” and “Do not drive a motor vehicle or operate heavy machinery while using marijuana.”^13^ The State of Washington mandates similar warnings and adds the admonishment to keep cannabis out “of the reach of children.”^14^ Oregon requires “poisoning” and “pregnancy” warning posters at dispensaries.^15^

The American Medical Association recommended warnings about risks to fetuses and breastfed babies from the use of cannabis by mothers.^16^ In an attempt to generate movement toward mandated warnings, Malouff and Rooke^17^ presented warnings suggested by multiple world leaders in cannabis research. The six recommended warnings mentioned risks relating to driving, long-term physical health, fetuses, mental health, dependence, and adolescent development.

Now-dated studies found that some youths and adults perceive health risks relating to cannabis use, including lung cancer, mental problems, memory problems, and other respiratory diseases.^18–20^ More recent studies showed that youths are familiar with some risks of cannabis use but not others.^21,22^ The same may be true for older individuals.

Cannabis users have a unique perspective on risks associated with use. That perspective is based partly on their own experiences and on their observations of others using cannabis. Their perspective could add valuable ideas to those of experts and could be important for their own future use levels. We therefore set out to determine what warnings users would recommend. We were interested in what overlap would occur with expert recommendations and what additional warnings users would recommend.

## Method

### Participants

To recruit participants, we used Facebook postings, announcements in psychology classes, and a university media release. Our recruiting invitation stated that we sought individuals who were “at least 18 years old and have used cannabis at least once” to participate anonymously in a study on “Government required cannabis warnings: Warnings suggested by individuals who have used cannabis.”

The recruiting efforts led to 288 adults (146 men; 142 women), with a mean age of 33.9, *SD*=12.9, completing the study, including 257 individuals living in Australia, 9 in the United States, 8 in the United Kingdom, 6 in New Zealand, 2 in Canada, 2 in Sweden, and 1 each in Brazil, Indonesia, and Portugal, and 1 country not provided. The mean self-reported age at first use of cannabis was 17.6, *SD*=4.0. The individuals in the study participated voluntarily, with no compensation to them or to any recruiting organization. The study had approval from the university Human Research Ethics Committee. Participants indicated their consent by selecting as an option “I agree to participate.”

### Procedure

#### Data collection

Participants anonymously completed the online research questionnaire. The beginning of the study stated: “This research seeks suggestions from cannabis users for health/safety warnings relating to the use of cannabis. Governments that allow the sale of medical cannabis or recreational cannabis might consider these suggestions if they decide to mandate health warnings on cannabis packages.” The key question for participants was: “If a state or country that permits the sale of cannabis decides to require specific health or safety-related warnings on packages of cannabis, what warning would you suggest?” Participants also answered questions about their sex, age, age at first use of cannabis, and their level of use. The question about level of use had options (Table 1) similar to a question in prior research that asked about level of cannabis use.^23,24^ For analyses, we included only individuals who indicated that they had used cannabis.

**Table 1.** **Participant Levels of Cannabis Use (*n*=288)**

Self-reported best description of use level	*n* (%)
Used only once or twice	64 (22)
1–11 times a year at present	44 (15)
1–3 days a month at present	17 (6)
1–3 days a week at present	27 (9)
4–7 days a week at present	74 (26)
Prior regular use but now not using	62 (22)

#### Category formation and categorization of suggested warnings

Eight participants gave more than one suggested warning. We considered each warning separately for counting the number of suggested warnings in each category.

One of us (first author) coded the warning suggestions into content categories, and then another one of us (third author) evaluated the category labels and coded warnings. Through an iterative approach, two of us (first and third authors) created category titles that suited the warnings in a category, and we ensured that the warnings in a category fit the content of the category. At the end of the coding process, the same two of us agreed that (1) the category titles described the content in the categories, (2) that each category had a least 15 warnings in it that fit the category title, and (3) that warnings not assigned to a category did not form some additional content category. Categorizing suggested warnings into content groups led to six types of warnings represented by at least 16 individual warnings (over 5% of the 301 total suggested warnings). We placed into a seventh, miscellaneous, category those suggested warnings that did not fit any of the six main content categories.

A third one of us (second author) then independently categorized the suggested warnings into one of the seven categories. Cohen's kappa for inter-rater agreement regarding the seven categories of warning was 0.77, *p*<0.001. That kappa level can be described as showing substantial agreement, somewhat below the 0.80 to 0.99 level of almost perfect agreement, according to much-cited standards of Viera and Garrett.^25^

## Results

Table 1 shows the widely varying cannabis-use levels of participants. The list below includes in parentheses the total number of suggested warnings (of 301) in the category and representative samples of actual statements by users.

1. *Marijuana use creates a risk of harm to mental health and psychological functioning* (96)*.* “May induce psychosis.” “May cause paranoia.” “Smoking marijuana may exacerbate mental illness.”
2. *Don't drive or operate machinery while under the influence of marijuana* (50)*.* “Don't drive under the influence.” “Do not use while driving or operating heavy machinery.” “Don't drug drive.”
3. *Marijuana use can cause short-term physical side effects* (34). “May induce drowsiness.” “May cause dizziness and disorientation.” “This drug may cause feelings of nausea.”
4. *Use responsibly* (23). “Follow dosing instructions on package.” “Use responsibly.” “To be used in moderation.”
5. *Marijuana use can cause serious long-term negative physical effects* (22). “Smoking marijuana is dangerous to your health.” “Smoking harms lungs.” “Heavy marijuana use is not good for the developing brain.”
6. *Marijuana use may lead to dependence, addiction, or abuse* (18)*.* “May be addictive.” “May be habit forming.” “May cause dependency.”

Four users mentioned risk of harm to adolescent brain development, a risk mentioned by multiple experts in the Malouff and Rooke study.^17^ We placed these four warnings in the category of serious long-term negative physical effects.

Two warnings suggested by experts in the study of Malouff and Rooke^17^ had no mention by the users: fetal harm risk and heart disease risk.

To examine whether sex, age, age at first use, or level of use was associated with suggesting warnings in particular categories, we used the phi coefficient to assess associations between warning category and each of the following variables: sex, age (divided at the median into under 35 or 35 and over), age at first use (divided at the median into under 18 or 18 and over), level of use (divided at the median into 3 or fewer days a week on average or more frequent use), and basis for suggesting the warning. To avoid violating the assumption of independence of observations, we used only the first warning suggested by the eight individuals who suggested more than one warning.

All associations were nonsignificant: sex, phi=0.18, *p*=0.15; age, phi=0.20, *p*=0.08; age at first use, phi=0.16, *p*=0.35; use level phi=0.192, *p*=0.10, except for basis of warning, phi=0.42, *p*<0.001. The result for basis of warning indicated that the responses for all warning categories were disproportionately based on personal experience and observations, rather than on something participants heard or read or on some other basis. Together, personal or observed experience was the basis for 77% of suggested warnings. Table 2 shows the basis for the suggested warnings.

**Table 2.** **Basis for Suggested Warnings (*n*=288)**

	No. of users stating this as basis for warning			
Warning	Own experience	Observed others	Heard or read about	Other
Harm to mental health or psychological functioning	52	20	11	11
Do not drive	30	6	6	6
Short-term physical side effects	21	5	1	0
Use responsibly	12	6	3	2
Long-term physical effects	5	3	11	2
Dependence	7	7	2	1
Other	42	6	4	6
Total	169 (59%)	53 (18%)	38 (13%)	28 (10%)

## Discussion

The results of this qualitative study overlap to some extent with warning recommendations of cannabis researchers. Like the cannabis users in the present study, experts recommended warnings relating to driving, mental health risks, long-term physical health risks, and dependency.^17^ However, the users suggested two types of warnings not in the list of warnings recommended by experts^17^ and not mentioned in a recent review of risks of cannabis use^7^: The first was *marijuana use can cause short-term physical side effects,* including dizziness, nausea, and drowsiness. This warning is similar to warnings on many medications. There is research evidence showing that cannabis use can lead to dizziness, nausea, and drowsiness.^26^ The second additional type of warning was *use responsibly*. Although the United States does not include such advice on its mandated warnings of alcohol containers, this type of mandated warning is common with regard to alcohol containers in other countries.^27^

When the user-recommended warnings are combined with expert-recommended warnings, the following eight warnings result: (1) *Don't drive or operate machinery while under the influence of marijuana.* (2) *Marijuana use creates a risk of harm to mental health and psychological functioning.* (3) *Marijuana use may lead to dependence, addiction, or abuse.* (4) *Marijuana use can cause serious long-term negative physical effects, including heart and lung disease*. (5) *Marijuana use can cause short-term physical side effects.* (6) *Use marijuana responsibly*. (7) *Marijuana can have negative effects on adolescent development. (8) Marijuana use can cause fetal harm.*

The cannabis experts in the study of Malouff and Rooke^17^ also recommended warnings relating to heart disease and fetal harm. Not a single user mentioned those risks. It could be that cannabis users are not as aware of these risks of cannabis use as with other risks.

The mandated cannabis package warnings in Colorado and Washington State^13,14^ mention two of the eight content areas: driving under the influence of cannabis and fetal harm. Six other content areas in our suggested list of warnings are not covered.

The current findings are consistent with prior findings that some youths consider cannabis potentially harmful and addictive.^21^ Warnings might increase the number of individuals who perceive cannabis use as potentially harmful.

The six categories of warnings suggested by participants overlap to some extent with existing mandated warnings in some countries for alcohol and tobacco products. In Australia, for example, mandated tobacco warnings^28^ (Australia Department of Health, 2016) mention serious health risks involving heart disease, lung disease, stroke, and cancer; they also mention fetal harm. In the United States, the mandated alcohol warnings mention risks of driving while intoxicated in addition to “health problems” and fetal harm^29^ (U.S. Code of Federal Regulations, 2015). The precedent for mandating warnings of this type has been set.

Various types of studies have indicated that warnings on alcohol and tobacco products increase awareness of risks and are associated with decreased use.^10,11^ Hence, the potential exists for cannabis warnings to have positive effects on risk-awareness and use level, possibly reducing harm caused by cannabis use.

The present study had a limitation in that most of the participants lived in Australia. It is unclear whether the current findings would be different with participants living in much different cultures. The study participants included an even split of men and women and a wide range of ages and use levels. Type of warning suggested was not significantly associated with any of these participant characteristics. These characteristics of the sample and findings may make the study more generalizable than it would be otherwise.

In sum, the present findings expand the range of potentially useful warnings beyond those recommended by experts. Governments that allow legal sale of cannabis could use the combined recommended warnings of experts and users in developing warnings to mandate on packages of legally sold cannabis. Furthermore, legal cannabis sellers could include the warnings on packages even without being legally required. They might include warnings either to serve the public interest or to reduce their risk of legal liability if a purchaser suffers some catastrophic harm as a result of use.^30^

Future research might examine several matters related to cannabis warnings. First, researchers could gather user-suggested warnings in various cultures. Cannabis users in countries such as the United States with substantial levels of cannabis legalization could be the most important individuals to sample. In much different cultures, the suggested warnings might be different. Second, future studies could test cannabis users' awareness of specific health and safety risks associated with use of cannabis. Low rates of awareness of particular risks might indicate the need for specific warning content. Third, researchers could assess the extent to which users find personally relevant the warnings suggested by users in the present study. Warnings are not likely to have positive effects if users do not believe the risks mentioned apply to them. Finally, studies could examine the effects of cannabis warnings on risk-awareness and use. Positive effects might include an increase in risk awareness; a decrease in leisure use; use of less dangerous delivery methods, such as vaporizers^31^; and use at less dangerous times, for example, not before driving.
